# A plea for minimally biased naturalistic philosophy

**DOI:** 10.1007/s11229-017-1628-0

**Published:** 2017-11-30

**Authors:** Andrea Polonioli

**Affiliations:** Department of Philosophy, University of Birmingham, 3 Elms Road, Edgbaston, Birmingham, B15 2TT UK

**Keywords:** Cognitive bias, Heuristics, Naturalism, Systematic review, Narrative review, Methodology

## Abstract

Naturalistic philosophers rely on literature search and review in a number of ways and for different purposes. Yet this article shows how processes of literature search and review are likely to be affected by widespread and systematic biases. A solution to this problem is offered here. Whilst the tradition of systematic reviews of literature from scientific disciplines has been neglected in philosophy, systematic reviews are important tools that minimize bias in literature search and review and allow for greater reproducibility and transparency. If naturalistic philosophers wish to reduce bias in their research, they should then supplement their traditional tools for literature search and review by including systematic methodologies.

## Introduction

Naturalism has been a hugely successful research program in contemporary analytic philosophy. Naturalistic philosophers have been following Quine (1952) in challenging the separation of philosophical from scientific research and arguing that philosophy and science are best conceived of as engaged in essentially the same enterprise, pursuing similar goals and deploying similar methods.

Here, it will be argued that there would be benefits for naturalistic philosophers if they expanded their methodological toolkit. The tools discussed here are the systematic methodologies for literature search and review that are widely employed in the natural, life and health sciences.

More in detail, the paper presents and defends the following claims. First, naturalistic philosophers do not philosophize in a vacuum and, in fact, rely on literature search and review in a number of ways and for several purposes. Second, biases and cognitive limitations are likely to affect literature search and review in many critical ways. Over the past decades, psychologists have described numerous ways in which judgment formation and information search can be biased, and there are no reasons to doubt that also literature search and review should be biased in important ways, and even in the field of philosophy. Third, scientists have come to widely adopt systematic reviews to minimize bias in the activity of literature search and review, and these tools should also be of wide interest to naturalistic philosophers. More precisely, systematic research review is a highly structured approach to cumulating knowledge. Progress in knowledge acquisition is the result of the integration of efforts, and literature reviews are vehicles for summarizing research. For systematic reviews, a clear set of rules exists for searching studies and for determining which should be included in or excluded from the analysis. The reproducibility of an experimental result is a fundamental assumption in science and in a similar fashion systematic reviews aim to allow for high reproducibility of conclusions by minimizing bias and maximizing transparency.

In the remainder of this paper these claims will be carefully discussed, and then combined to offer a plea for a type of minimally biased philosophy: if naturalistic philosophers wish to reduce bias in philosophy, as it is here assumed that they should, then they should consider ways to supplement their traditional tools for literature search and review by including systematic reviews.

A few remarks are in order here. Interestingly, a number of researchers have raised methodological concerns relating to the ones examined here (Steel et al. 2017; Machery 2016; Machery and Cohen 2012; Faust and Meehl 2002), focusing more specifically on the limitations of the case-study method in the context of the philosophy of science. In light of their concerns, some of these researchers have also ventured to recommend the use of alternative and quantitative methods (Machery 2016), such as those used in the field bibliometrics, and which often involve formal tools. These are valuable recommendations and some philosophers have already offered interesting applications of quantitative tools from bibliometrics (e.g., Wray and Bornmann 2015). Still, it seems indeed quite surprising that among the several tools proposed to complement the philosophers’ toolbox systematic reviews have not as yet been considered. The systematic methodologies advocated here are meant to offer tools for wide use in the philosophical community. As shown in this essay, these methodologies help minimize bias and are applicable to a broad range of questions.^1^

The wide applicability of systematic reviews invites some further considerations. Specifically, this essay recommends the use of systematic reviews within naturalistic philosophy, but there are also reasons to think that the concerns discussed here do affect other philosophical approaches as well, which could also benefit from a more systematic approach to literature search and review. After all, systematic reviews have already been applied to non-empirical literature in the humanities and social sciences (Strech and Sofaer 2012; McDougall and Notini 2013), suggesting that a suitable model of systematic review could in principle be applied to broad areas of philosophical inquiry. Still, two considerations seem to justify the choice to focus more narrowly on naturalistic philosophy in this paper. First, as naturalistic philosophers see science and philosophy as part of the same enterprise, they are also plausibly more likely to listen to the concerns examined here and to import tools from scientific disciplines. Second, it is generally clearer how to apply systematic tools and methods to surveys of empirical results, and hence recommendations can be more concrete and specific in this case.^2^

This essay is organized as follows. Section 2 discusses the ways in which naturalistic philosophers rely on literature search and review. Section 3 argues that processes involved in literature search and review are likely to be biased in non-trivial ways. Section 4 compares narrative and systematic approaches to literature search and review and discusses the virtues of the latter approaches. Section 5 strengthens the case for the adoption of systematic methodologies by naturalistic philosophers and offers some practical recommendations concerning research, publishing and training practices. Finally, Sect. 6 delivers the conclusion.

## Naturalistic philosophy and literature review

A hot topic in metaphilosophy concerns how best to describe the methods used by philosophers and their practices. Many of the recent discussions on this topic have focused on whether, to what extent, and how analytic philosophy rests on the use of intuitions (e.g., Cappelen 2012; Andow 2016). Still, we should not underestimate the importance of literature search and review for the philosophical profession, at least in many areas of philosophical investigation.

Arguably, philosophers’ reliance on literature search and review is particularly evident in the case of naturalistic philosophy.^3^ Naturalism comes in different forms, and a distinction is often made between experimental and empirical philosophy (Prinz 2008; Rose and Danks 2013). Experimental philosophy has recently adopted methodologies from various sciences—typically behavioural, social and cognitive sciences—and engaged in a number of empirical projects to answer philosophically interesting questions (Knobe and Nichols 2008; Alexander 2012; Sytsma and Buckwalter 2016; Sytsma and Livengood 2015). Empirical philosophers use instead in their philosophical theorizing empirical results acquired by professional scientists. More precisely, they search for, screen and cite relevant empirical research outputs. As Jesse Prinz put it, “empirical philosophy works by citation” (2008, p. 200). What matters, here, is that this characterization of empirical philosophy seems to capture a great deal of work in naturalistic philosophy; if we asked what naturalistic philosophers actually do when they carry out philosophical research, a plausible answer could not help but mention their engagement with literature search and review as an important aspect of it.

Still, there are different types of activity that require a thorough literature review to be conducted, and different uses of literature review exist. Naturalistic philosophers use empirical findings to support their claims and premises. They also often contribute to scientific theorizing by providing novel hypotheses, synthesizing swathes of empirical and theoretical works, and suggesting empirical research. Most typically, naturalistic philosophers cite neuroscientists and psychologists, but they also call on linguists, evolutionary biologists, roboticists, and anthropologists, as well as physicists or chemists.

There are plenty of well-known works from naturalistic philosophers that can exemplify these uses. Yet mentioning a few paradigmatic examples might be helpful here. For instance, philosopher Clark (2013) extensively surveyed scholarly work to argue that brains are fundamentally prediction-error minimizing devices trying to self-generate the sensory streams that are currently arriving from the world. Further, philosophers have also appealed to empirical research to diagnose problems affecting their research community and ameliorate the prospects for philosophical research. In particular, Saul (2013) linked the topic of underrepresentation of women in philosophy to empirical research on implicit bias. She stresses that ‘over the last few decades, psychologists have established very clearly that human beings, even those who hold strongly egalitarian ideals, are prone to a range of unconscious biases against members of groups that are stigmatized in certain areas’ (p. 243). Moreover, Prinz (2004) has offered an up-to-date version of William James’s theory of emotion that he takes to be well supported by the wealth of evidence from empirical psychology and neuroscience that he discusses. Still, whilst literature on cognitive science has garnered a huge deal of attention within naturalistic philosophy, there are plenty of other empirical subjects that have direct relevance to philosophical theorizing. For instance, philosophers have looked at quantum mechanics to inform their discussions over free will (e.g., Loewer 1996; Hodgson 2002) or consulted literature from physics (e.g. Leslie 1982, 1992) to draw considerations about morality, evolution or cosmology connected to the anthropic principle (Carter 1974). The work of other philosophers has instead drawn heavily from research in economics and, more generally, in the social sciences (e.g., Guala 2005; Reiss 2016).

Notably, literature review can also constitute an original and valuable piece of research in itself. Review articles come in many trades and different taxonomies are available as well: they can be critical, literature, mapping, generic, qualitative, rapid, scoping, state-of-the-art, systematic, systematized, and umbrella reviews (Grant and Booth 2009). Some classifications follow instead a pragmatic approach. For instance, the ISI Web of Knowledge Science Citation Index categorizes a paper as a review if it either (1) contains more than 100 references; or (2) appears in a review journal or the review section of a journal; or (3) states in the abstract that it is a review.^4^

Review articles are published in philosophy journals too. For example, the journal *Philosophy Compass* publishes original, peer-reviewed survey articles of the most important research from across the entire discipline. In its section on Naturalistic Philosophy it is possible to find entries on topics such as the “Experimental Philosophy of Aesthetics” (Cova et al. 2015) or “Causation: Empirical Trends and Future Directions” (Rose and Danks 2012). Rather than providing a basis for the researchers’ own endeavors, this type of literature review creates a solid starting point for all other members of the community that are interested in a particular topic, and who can refer to these reviews to support some of their claims. Further, if philosophers wish to have impact outside their field too, it becomes advisable to have review articles that present the state of the art on a particular philosophical topic and the main results, so that scientists, policy makers, or any member of a particular profession can more easily become acquainted with the status of a philosophical debate and avoid taking onboard questionable assumptions in their practice. Such review articles can also prove to be useful tools for teaching purposes, providing students with a balanced treatment of a particular topic.

## Heuristics and biases in literature search and review

So far, it has been suggested that naturalistic philosophers rely on literature search and review in their philosophical work. In light of this, however, it will now be shown how naturalistic philosophers also seem to face a number of hurdles. More precisely, here it is argued that biases can in fact compromise the reliability of processes of literature search and review. Section 3.1 introduces some of the possible relevant heuristics and biases. Section 3.2 discusses heuristics and biases in relation to the growing number of research outputs available in the literature. Finally, Sect. 3.3 discusses and rebuts a possible objection.

### The impact of biases on search and review

Naturalistic philosophers need to carefully consider the fact that literature search and review are likely to be constrained by cognitive limitations and vulnerable to biases. Scientists sometimes warn of these risks. For instance, Roy Baumeister wrote that:

Although literature reviews are less subject than empirical investigations to capitalizing on chance, they are probably more susceptible to the danger of confirmation bias. Many good literature reviews involve seeing a theoretical pattern or principle in multiple spheres of behavior and evidence, and putting together such a paper undoubtedly involves an aggressive search for evidence that fits the hypothesized pattern (1997, p. 319).

These considerations are not at all untethered. As it turns out, over the past five decades psychologists have documented many ways in which our reasoning and decision-making can be systematically biased by cognitive, motivational and affective factors (Hastie and Dawes 2010). People have been shown to deploy heuristics that in several contexts lead to a number of biases and, in turn, poor or ill-grounded decision-making.

Philosophers have been hugely interested in this psychological literature (Samuels et al. 2002; Lee et al. 2013; Saul 2013; Polonioli 2014). Still, a point that has not been properly acknowledged in the philosophical literature is that there are also good reasons to think that biases and cognitive limitations might generally have a huge and negative impact on literature search and reviews. After all, literature search and review are just a specific case of information search and related judgment and decision-making, where the latter have been described as riddled with biases. In brief, scholars and scientists typically search for relevant information, process it, and form a number of judgments about the information reviewed. Arguably, the same processes that underlie many other instances of information search and assessment are likely to be at work on these occasions as well.

Search for evidence can be biased in critical ways. An obvious and well-known bias is the abovementioned *confirmation bias*, which consists in the “seeking or interpreting of evidence in ways that are partial to existing beliefs, expectations, or a hypothesis in hand” (Nickerson 1998, p. 175). When people seek new information, their information search processes are often biased in favor of the seekers’ previously held beliefs, expectations, or desired conclusions. For example, people have been shown to favor information that supports their social stereotypes (Johnston 1996), attitudes (Lundgren and Prislin 1998), expectations in negotiations (Pinkley et al. 1995), and self-serving conclusions. Arguably, if a researcher is carrying out a literature review on the positive psychological effects of holding a particular sort of belief, such as believing in free will, confirmation biases might result in her ignoring undesired information and her focusing on what seems to support her previously held belief.

Moreover, consider research on *implicit biases*, where the latter are typically understood as ‘largely unconscious tendencies to automatically associate concepts with one another’ (Saul 2013, p. 244). Whilst there are still a number of open questions regarding the nature and frequency of implicit biases, what seems to be supported by copious evidence is that decision makers are often biased by people’s gender, ethnic background, and sexual preference, for example when they select job candidates based on CVs, and also by people’s appearance (especially weight and height in relation to gender) when they interview job candidates (Steinpreis et al. 1999). If these biases can result in judging members of stigmatized groups negatively, it is not difficult to see how they could impact on the search and assessment of scholarly information. Research published by researchers from particular groups might be just ignored or its value might be slighted.^5^

Further, consider how people have been shown to heavily rely on what is most salient or available to them in their judgment and decision-making (Tversky and Kahneman 1973). In particular, recent occurrences, because they are cognitively more salient, often lead people to misrepresent the probability of certain types of events. For instance, “the subjective probability of traffic accidents rises temporarily when one sees a car overturned” (p. 11). People are apt to accept one of two competing views merely because they heard evidence supporting the one view more recently (Kahneman and Tversky 1982). In light of this, it is not unlikely that researchers trying to organize findings in support of a particular hypothesis will rely on partial, although quite salient or easily accessible, information.

Finally, researchers attempting to deliver an evenhanded treatment of the literature are supposed to discuss which views are more and less widely shared. But evidence from several studies suggests that social observers tend to perceive a form of *egocentric bias* with respect to the relative commonness of their responses. The best-known example comes from a 1977 study in which Ross, Greene and House asked students to walk around a campus wearing a sandwich board with the word ‘repent’ on it. Students could agree to wear the board, doing the experimenters a favor, or disagree and participate in a later study. Those who offered to wear the sign (50%) estimated that more than half of their peers would also agree to do so (average estimation 63.5%). Those who declined thought that about a quarter of those asked would accept (average estimation 23.3%). Apparently, students overestimated how similar others’ preferences were to their own. These findings might be taken to suggest, for example, that researchers trying to assess the popularity of a particular view or account might produce inaccurate assessments of its popularity (Gilovich 1990).

Overall, confirmation biases, salience and availability biases, as well as false consensus biases are among the many effects and distortions that might impact on literature search and review. There are no compelling reasons to assume that naturalistic philosophers would be immune from such biases. After all, it seems that everybody is affected by biases to some degree, irrespective of factors like general intelligence or open mindedness (Stanovich and West 2008). Though expertise in specific domains might have positive effects on judgment and decision-making, some findings indicate that experienced professionals often display either roughly the same biases as college students or the same biases at somewhat reduced levels. More precisely, psychological research has demonstrated that a wide variety of biases often affect the assessments that many professionals, including physicians, investors, accountants, option traders, real estate agents, engineers, and psychologists, are trained to make (e.g., McNeil et al. 1982; Choi and Pritchard 2003; Bazerman et al. 2002; Fox et al. 1996). More recently, it has been shown that also philosophers tend commit the very same biases (Schwitzgebel and Cushman 2012, 2015; but see also Livengood et al. 2010). In light of this, it seems highly plausible that when naturalistic philosophers search for and assess scholarly content, they can be affected by these biases.

But there is also more direct evidence on the impact of biases in scholarly contexts. For instance, Roumbanis (2017) explored *anchoring effects*, whereby the first numerical value that an individual encounters tends to influence his or her judgment of what is to be assessed, in the process of peer reviewing research proposals. Yet there have also been studies on biases in the process of literature selection and assessment. For instance, literature on biases in literature selection has suggested that factors such as the reputation and prominence of an author could influence citation decisions (the so-called “Matthew effect”; Merton 1968). More recently, focus has been placed on testing whether female scholars are systematically denied credit for their work (the so-called “Matilda effect,” Rossiter 1993). In the case of Matilda effects, unconscious automatic processes are assumed to trigger gender bias. In light of this, an author’s gender indirectly influences citation behavior, as it functions as a simple cognitive heuristic to assess scientific quality, challenging the assumption that citations represent payments of intellectual debt and, to that end, would strictly follow the criteria of relevance and quality (cf., e.g., the discussion in Baldi 1998). There is growing evidence on the impact of such biases in scholarly contexts (cf. Lincoln et al. 2012; Potthoff and Zimmermann 2017).

### “Big science” and heuristics

As it turns out, in the context of literature search and review reliance on heuristics seems quite likely to occur, also because of the increasing and huge amount of research materials published. Herbert Simon claimed that ‘human rational behavior is shaped by a pair of scissors whose blades are the structure of task environments and the computational capabilities of the actor’ (1990, p. 7). Whilst an important generalization that comes out of efforts to study human information processing is that an individual is a limited information processing system (Newell and Simon 1972), it is also the case that researchers have to face a rather complex environment in the case of literature search and review, as the relevant literature is huge and the database of papers and noteworthy content is not only massive but also growing fast, as explained by Derek De Solla Price in his “Little science, big science...and beyond” (1986) (see also Génova et al. 2016). Specifically, the volume of research available in most fields is expanding rapidly and there has been an increase in the rate of published outputs, although it is less clear to what extent this might reflect an increase in knowledge accumulation or rather a tendency to “slice” one research project into too many papers (i.e., “salami publishing”). What is clearer, instead, is that in light of people’s memory and cognitive limitations, recall of huge amounts of relevant information and literature might be far from optimal. Moreover, in light of people’s computational limitations, analyses of huge databases of scholarly content might also be far from optimal.

Clearly, not all of the processes interfering with the reliability of outcomes of literature search and review need to be unconscious. In addition to the abovementioned unconscious biases, naturalistic philosophers could also be deliberately adopting questionable strategies in processing the literature, resulting for instance in more favorable treatment of close colleagues. Yet unconscious biases are particularly worrisome, as they are hardly detected by the agent. Correction of distorting factors seems harder to occur than one might think, and it has recently been suggested that rationalization of biased choices or judgments might be common in the work of philosophers and scientists as well (Schwitzgebel and Ellis 2016).

### How smart can simple heuristics be?

A possible objection needs to be addressed, though. To be sure, heuristics do not just lead to critical biases. In some contexts, people have also been shown to deploy heuristics that enable them to gather and assess information effectively (Gigerenzer 2000).^6^ Fast-and-frugal heuristics that take into account only few cues and little of the available information might lead to accurate predictions and estimates in a number of contexts, and such adaptive heuristics might also be at work in the context of literature search and review. This is an important point. Yet, whilst it is important not to overlook this more positive view of our decision-making performance (Robins and Craik 1993; Christensen-Szalanski and Beach 1984; Lopes 1991), such acknowledgment should not be read as being at odds with the recognition of the impact of the abovementioned biases and their problematic nature. In addition, it is also unclear to what extent the most plausible heuristics at work in this context could be *successful*. The most prominent cues to be used as proxies for the value of the relevant content are likely to be the number of articles’ citations, the Journal Impact Factor, or similar metrics. But it can take long time for an article to accumulate citations, especially in the humanities, meaning that it is hard to use such number as a cue to identify relevant published material. Further, whilst it is sometimes argued that social media activity provides useful indications for the future citations, recent research suggests that altmetrics are best conceived of as measuring a different kind of research impact (Erdt et al. 2016). Moreover, the view that Journal Impact Factor is a reliable indicator of the quality of journals has been heavily criticized (e.g., Moustafa 2014) and a number of authors have pointed out that it does not necessarily correlate with several aspects of the journal’s quality (Brembs et al. 2013).

Overall, there are good reasons to carefully consider the heuristics and biases that might be operating in the context of literature search and review. Because of these factors, and especially in an environment characterized by growing scholarly production, researchers might find it difficult to process information in a way that serves well and furthers goals of accuracy and truth. But if common research practices do not serve these goals well, then this should look like a worrying situation for researchers. The question arises as to what options are available to remedy the situation described above. Arkes et al. (2006, 2010) referred to work on the inaccuracy of judgment and decision-making (e.g., Dawes et al. 1989) to urge that we should “examine the benefits of a more routinized, mechanical method for evaluating scientific materials such as research presentations at professional conventions or proposals submitted to federal funding agencies” (2006, p. 430). The next section examines precisely ways to improve the reliability of the survey of research materials in the context of literature reviews by appealing to systematic methodologies.

## Narrative and systematic methods for literature review

Biases threaten the reliability of literature search and review. But different types of search and review differ in terms of vulnerability. Section 4.1 characterizes narrative methods for literature search and review, which are typically used in philosophy. Section 4.2 introduces the framework of systematic reviews. Section 4.3 discusses different formats and common criteria in systematic reviews. Section 4.4 addresses and rebuts some possible objections to the use of systematic reviews.

### Beyond purely narrative approaches

In philosophy, as well as in many other fields in the humanities, literature search and review is typically “narrative” in character (but see Feltz and Cova 2014), whereby a content expert writes about a particular topic offering a comprehensive narrative synthesis of previously published materials, usually not describing the methods deployed and the criteria for inclusion and review of the literature. In brief, the authors of narrative reviews are free to include and exclude research as they like, and they are free in their evaluation of research too. For instance, in her synthesis of the literature on framing effects, Joanna Demaree-Cotton writes the following: “I have included all relevant studies of which I am aware” (2016, p. 9). In this case, the author is explicitly acknowledging the lack of objective selection criteria in her study. More commonly, however, the nature of the criteria for inclusion and methods for literature search and selection is not even addressed in published papers within philosophy. Consider, for example, a paper recently published in *Philosophical Psychology*, in which Smithdeal (2016) reviewed empirical evidence allegedly suggesting that belief in free will is beneficial. His review was narrative in character: the author appealed to some sources suggesting that belief in free will offers valuable support for prosocial behavior (Vohs and Schooler 2008; Baumeister et al. 2009), and then criticised some studies pointing to possible detrimental effects of belief in free will (Nadelhoffer and Tocchetto 2013). Notably, failure to disclose selection strategies and decisions might in fact matter quite a lot here. Besides the referenced sources, which suggest that disbelief in free will is linked with a decreased willingness to help others, some other studies examining how free will beliefs influence true self-knowledge (Seto and Hicks 2016) seem to be highly relevant to the research question addressed by Smithdeal’s paper. It is hard to tell, however, if the author was aware of the existence of this study and, in case he was, why or how such sources were excluded. Further, relevant studies have been published after the publication of Smithdeal’s paper. Notably, Caspar et al. (2017) provided further evidence that disbelief in free will had a positive impact on the morality of decisions toward others. Would this paper feature in the author’s analysis, should he be writing it today? Being unable to answer this question seems to represent a limitation of current narrative methodologies. As this example might reveal, popular narrative methods for search and review seem to offer room for biases and cherry picking, and reviews which use these approaches risk being subjective and hardly replicable.

### Systematic reviews and bias minimization

Interestingly, outside philosophy it has been frequently pointed out that traditional narrative reviews, in spite of some clear benefits, are also prone to error and bias. More precisely, whilst some researchers have suggested ways to reduce bias in literature search and review by improving traditional narrative approaches to review (Baumeister 2013), many others have argued that more rigorous and unbiased types of analysis should be offered to replace traditional narrative reviews (Kitsiou et al. 2013; Templier and Parè 2015). In particular, in the 1970s and early 1980s, scientists started to draw attention to the systematic steps needed to minimize bias and random errors in reviews of research (Light and Smith 1971; Glass 1976; Rosenthal 1978; Jackson 1980; Cooper 1982). In this context, scientists appealed to systematic reviews as a useful tool to navigate through complex bodies of literature and summarize them in a way that reduces bias. In the huge literature on systematic approaches to literature review, the adjective ‘systematic’ is typically contrasted with ‘haphazard study selection procedures’ or even ‘arbitrary study selection procedures’ (Slavin 1986, p. 6). It should be noted, however, that systematic reviews should also be distinguished here from meta-analyses: only when results are mathematically combined (a process sometimes referred to as pooling), this is referred to as meta-analysis. As the Cochrane Collaboration Handbook points out, in the case of systematic reviews “statistical methods (meta-analysis) may or may not be used to analyse and summarise the results of the included studies” (Higgins and Green 2011).^7^

The appeal of systematic reviews varies from field to field. In the health sciences, systematic reviews have now become a standard and are well understood by all contemporary practitioners. Three decades ago, Mulrow et al. lamented hat “medical reviews are often subjective, unsound and inefficient”, and that “strategies for identifying and selecting information are rarely defined” (1987, p. 485). But things changed significantly in the following years (Bracken 2001). In other fields, instead, systematic reviews are still not mainstream. For example, consider the field of psychology. Narrative methods of review have been dominant and widely taught for long time. Some researchers encouraged authors of reviews “to take a point of view based on theory and offer readers a point of view that integrates the review” (Sternberg 1991, p. 3). Currently, some journals, like *Psychological Bulletin*, increasingly are publishing systematic reviews, while others, such as *Trends in Cognitive Sciences,* still publish narrative reviews only, as does the *Annual Review of Psychology *too.

### Formats and criteria for systematic reviews

Systematic reviews often address a question formulated in the Participants (Population), Intervention, Comparisons, Outcome (PICO) format. The question identifies a population, the intervention being investigated, a comparison point or points to the intervention, and the outcome of interest (Higgins and Green 2011, section 5.1.1). For example, a researcher might ask, “for older adults with musculoskeletal disorders, is home-based rehabilitation more effective than inpatient rehabilitation in relation to function, cognition and quality of life?” (Stolee et al. 2012). Within this framework, questions might be quite diverse in their nature, and in the medical field they might for instance seek to explore possible harm (e.g., will there be any negative effects?), prognosis (e.g., what is the likely outcome of this problem?), or etiology (e.g., what causes this problem?) of a particular effect. Further, systematic reviews may ask broader or narrower questions, and it is generally important to strike a balance between comprehensiveness and precision when developing a search strategy for a question. In brief, increasing the comprehensiveness of a search might result in reducing its precision and retrieving more irrelevant articles.

Systematic review methods often use peer-reviewed and published protocols to lay out the methods for a review: searches for studies, articles screening for relevance and quality, and data extraction and synthesis are typically undertaken according to a predetermined strategy. Different protocols for systematic reviews are typically followed in different disciplines. Methods in environmental sciences are outlined by the Collaboration for Environmental Evidence, in social sciences by the Campbell Collaboration and in medicine by the Cochrane Collaboration.

It is interesting to note that in several contexts, and especially outside the health and biomedical sciences, the PICO model has appeared to be too narrow and strict. For instance, Strech et al. (2008) tried to offer a model of systematic reviews for empirical bioethics, which is a field that heavily relies on interviews studies. Strech et al. advocated a model based on Methodology, Issue, Participants for review questions over the PICO format (2008, p. 473). The authors proposed a model based on 7-steps for systematic reviews of empirical bioethics: (1) careful definition of review question; (2) selection of relevant databases; (3) application of ancillary search strategies; (4) development of search algorithms; (5) relevance assessment of the retrieved references; (6) quality assessment of included studies; and (7) data analysis and presentation. Attempts such as this one suggest that it is indeed possible to pursue systematic reviews even in cases where the PICO model appears to be too narrow.

Yet, even in the biomedical and health sciences equal emphasis on each component of PICO is not necessary. For example, Shumway-Cook et al. (1997) address “the effect of multidimensional exercises on balance, mobility, and fall risk in community-dwelling older adults”, referring to a population, outcome and effect, but do not state a comparison in their question. Furthermore, systematic reviews of definitions and operationalizations of concepts and notions used in biomedical and health sciences have recently been offered, whereby such studies clearly depart from the PICO model described above (e.g., Bruce et al. 2001; Hajarizadeh et al. 2012). For example, Sørensen et al. (2012) seek to systematically address definitions and conceptualizations of the concept of health literacy.

This discussion should show how the systematic approach to literature search and review has been implemented in different ways in different contexts. Importantly, however, irrespective of the specific layout of the systematic review, the criteria used to select studies for inclusion should be clearly stated, alongside the bibliographic databases searched, the dates and periods searched and any constraints, such as language. More precisely, in addition to describing the search strategy, selection and data collection process, systematic reviews should also clearly discuss their objectives. In brief, systematic reviews seem to differ from traditional narrative reviews by virtue of being:

Systematic/organized: Systematic reviews are conducted according to a system or method that is designed in relation to and specifically to address the question the review is setting out to answer.

Transparent/explicit: The method used in the review is explicitly stated.

Replicable/updatable: As with many forms of primary research, the method and the way it is reported should be sufficiently detailed and clear such that other researchers can repeat the review, repeat it with modifications or update it.

Synthesize/summarize: Systematic reviews pull together in a structured and organized way the results of the review in order to summarize the evidence relating to the review question.

As it turns out, whilst systematic reviews might follow different protocols and focus on somewhat different questions, they are nevertheless supposed to incorporate a set of key principles of scientific methodology and depart from traditional narrative approaches to literature review. For instance, Cooper nicely expresses the spirit behind the systematic review movement in the introduction to his book, Synthesizing Research:

The approach to research synthesis presented in this book represents a significant departure from how reviews had been conducted just 20 years ago. Instead of a subjective, narrative approach, this book presents an objective systematic approach. Here, the reader will learn how to carry out an integration of research according to scientific principles and rules. The intended result is a research synthesis that can be replicated by others, can create consensus among scholars, and can focus debate in a constructive fashion (Cooper 1998, p. xi).

Finally, an important aspect to highlight is that systematic reviews are typically conducted in a team. Ensuring that tasks such as selection of studies for inclusion and data extraction can be performed by at least two people independently may increase the chance that errors and biases be detected. Importantly, at least when considering biases like confirmation, there is evidence suggesting that groups perform better than single individuals. More precisely, although groups in some contexts do fall prey to some of the errors made by the single individual, for many important biases such as confirmation, groups outperform individuals (Maciejovsky et al. 2013), and there is not evidence that single individuals outperform groups.

### Objections and replies

There are a few concerns and objections that need to be cleared up at this stage. A first worry one might have is that systematic reviews will actually fail to successfully address and neutralize the problematic impact of biases. On one hand, it is obvious that systematic methodologies do not completely eliminate subjectivity from the process of review. After all, when researchers try to operationalize a research question, they are still called to make some decisions. For instance, one still needs to define what counts as older population. On the other hand, systematic literature reviews are undertaken according to strict guidelines to minimize subjectivity, maximize transparency and replicability, and are supposed to provide a highly reliable review of evidence pertaining to a specific topic. The scientific method has the invaluable benefit of affording a systematic and unbiased investigation, and systematic reviews apply it to the practice of literature search of review. Systematic methods aim at making literature search and review objective: the reasoning is that subjectivity is a source of bias, and one that can and must be minimized by developing a clear protocol, making all the steps and the criteria explicit, following these steps and documenting all the relevant activity. By so doing, one is likely to maximize the chances of producing valid conclusions, and also makes the review replicable. It should be noted, here, that the use of the concept “objective” is eminently complicated, as also recent philosophical (e.g., Douglas 2004) and historical (e.g., Daston and Galison 2010) analyses demonstrate. But in general the objectivity of results is thought to be a consequence of the method being objective.

Still, some have expressed further qualms about using these methodologies. Whilst it is frequently argued that the “the use of explicit, systematic methods in reviews limits bias (systematic errors) and reduces chance effects, thus providing more reliable results upon which to draw conclusions and make decisions” (Higgins and Green 2011), one possible objection is that peer review would in any case wash out researchers’ biases, eventually leading to reliable surveys of the literature. This, however, seems to rely on too romantic a view of peer review, and one with several problems. Whilst peer review typically brings a measure of rigor and trust to scholarly communication, the reliability of peer-review is far from optimal, and several biases in peer review have also been identified (Lee et al. 2013; Lee 2015; Shalvi et al. 2010). There are well known cases of so-called Mendel syndrome, mentioned after Gregor Mendel, whose discoveries in plant genetics were so unprecedented that it took thirty-four years for the scientific community to catch up to it (Van Raan 2004; Gorry and Ragouet 2016). Moreover, obvious failures of peer review have also been clearly documented (Hawkes 2013). Even more importantly, the very fact that systematic reviews and narrative reviews have, at least in some cases, been shown to deliver results that are at odds suggests that pointing to peer review as a silver bullet might be an unwarranted move (Cipriani and Geddes 2003). For instance, De Dreu and Weingart (2003) show in a systematic review that the relationship between task conflict, team performance and team satisfaction is largely negative even though both academic papers and textbooks regularly report that task conflict has a generally positive effect.

Other critics of systematic reviews argue that a major threat to systematic reviews is dissemination bias, often referred to as publication bias, and which describes the selective publication and dissemination of results. In this situation, published studies constitute a biased sample leading to spurious conclusions. Published research can then be shaped by file-drawer effects (Rosenthal 1979). Again, this does not read as a knockdown objection, and at least for two reasons. First, whilst systematic reviews might not solve these problems, narrative reviews do not seem to be obviously better positioned at dealing with them. An argument would be needed to support this claim. Second, it actually seems that the methods of systematic reviews can be applied to the grey literature as well. For instance, these methods can be applied to doctoral dissertations as well as conference proceedings. In fact, Cochrane systematic reviews use very comprehensive search strategies and include both published and unpublished studies. Overall, publication bias is clearly an important problem that the research community and research gatekeepers need to address, but also one which is orthogonal to the debate over the merits of narrative and systematic reviews.

Where does all this lead us? The thrust of the section is not to argue that narrative reviews should be replaced by systematic reviews *tout court*. Arguably, narrative reviews have important benefits, including a broad overview of relevant information tempered by years of knowledge from an experienced author. It is also true that the narrative thread can be lost in the strict rules of systematic review, which might hinder the piece’s readability. As it turns out, the benefits of appealing to a particular approach might depend on the specific situation. For instance, Baumeister points out that in some cases:

A narrative rather than a meta-analytic review suits this purpose, in the interest of presenting a richer description of the prejudice-reduction literature. Moreover, the methods, interventions, and dependent variables are so diverse that meta-analysis is potentially meaningless (Baumeister and Leary 1997, see also Hafer and Bègue 2005), especially given that many of the research designs used in this literature are prone to bias, rendering their findings unsuitable for meta-analysis.

The point that this section seeks to drive home is that systematic approaches to review should at least be seen as important complements to traditional methods of literature search and review, as the former are better placed at reducing bias and increasing reproducibility. Although there is often some tension between the users of the two methods, and some experts who favour systematic analyses disdain narrative approaches as obsolete, both methods could actually have a valuable place in science.

## Systematic reviews for naturalistic philosophers

So far, it has been shown that systematic reviews are methodologies widely employed in natural, life and health sciences, that they offer important tools to minimize bias and increase transparency and reproducibility, and that they can come with different (more or less rigid) formats. Since naturalistic philosophers are also likely to be affected by critical biases in the process of literature search and review, it seems tempting to conclude that they should also carefully consider these tools. Notably, this outcome would be in line with recent claims put forward by experimental philosophers, which have suggested that philosophers should expand their toolbox to include a wide array of methods used in the sciences (Machery 2016; Machery and O’Neill 2014). This essay indeed recommends a wide application of these systematic tools: philosophers interested in appealing to empirical evidence in their analyses could benefit from the use of systematic methods, irrespective of whether such evidence is coming from research in physics, chemistry or cognitive science. However, there are a few outstanding tasks before this conclusion can be fully accepted. This section strengthens the case for systematic reviews in naturalistic philosophy by ironing out the details of the proposal on offer and addressing some possible concerns. More precisely, Sect. 5.1 shows why this plea for minimally biased naturalistic philosophy is especially timely and relevant. Section 5.2 defends the feasibility of systematic reviews within naturalistic philosophy. Section 5.3 addresses and rebuts a possible objection. Finally, Sect. 5.4 delivers practical recommendations concerning research, publishing and teaching practices.

### A timely call for systematic reviews

Naturalistic philosophers should find the case made here for the use of systematic methodologies to be particularly timely and relevant. Systematic methods have already been recently applied to address some topics and research questions that have attracted the attention of several philosophers. Notably, in such cases systematic methods have often played a seemingly corrective function, throwing doubt on some claims that are widely acclaimed in the literature, or just showing the need for further data to substantiate particular claims. This seems to suggest that, far from being an unnecessary complication in carrying out philosophical projects, systematic reviews might be powerful tools in the process of selecting and validating one’s evidence.

Consider one of the most important debates in philosophy and cognitive science, namely that concerning people’s (ir)rationality (e.g., Stich 1990; Stein 1996; Gigerenzer 1996; Kahneman and Tversky 1996; Samuels et al. 2002; Oaksford and Chater 2007; Todd and Gigerenzer 2012). One important question in this debate is whether people’s cognitive biases lead people to worse health, wealth and happiness (e.g., Sunstein and Thaler 2008; Bortolotti and Antrobus 2015; McKay and Dennett 2009; Polonioli 2014). As it turns out, these discussions have been taken to have important philosophical implications, for instance concerning the normative value of formal principles of rationality based on logic, probability theory and rationality decision theory (e.g., Larrick et al. 1993; Wallin 2013; Polonioli 2014; Boudry et al. 2016).

One frustrating aspect in the debate is that these discussions are typically carried out at quite an abstract level. Still, a recent innovation in the debate on rationality has been to appeal to more systematic methodologies in literature search and review. Specifically, Arkes et al. (2016) tried to address the relationship between biases and such outcomes by conducting “several systematic *Web of Knowledge *searches for the major coherence rules reported in the literature”, briefly outlining the search procedures and reporting, among other findings, “little evidence that coherence violations incur material costs” (Arkes et al. 2016, p. 22). Their findings led some scholars to conclude that:

Systematic literature searches show lack of evidence that these cognitive illusions, even if they existed, would cause actual harm in terms of less wealth, health, or happiness (Arkes et al. 2016; Berg and Gigerenzer 2010) (Mousavi et al. 2016, p. 281).

As it turns out, the study by Arkes et al. (2016) is by no means conclusive but should at least be seen as a small step in the right direction: by complementing narrative approaches with more objective, structured and systematic searches and reviews it is possible to push forward important debates and, where needed, correct any unwarranted claims. There have been other applications of systematic methods to address topics of interest to philosophers. For instance, philosophers have often embraced the assumption that emotions and affective processes cause moral judgment. Philosopher Joshua May writes that “scientists have apparently amassed converging evidence that emotions play a substantial role in the production of most, if not all, of our moral judgments” (2014, p. 125). But Landy and Goodwin (2015; see, however, also Schnall et al. 2015) offer a systematic review in which they consider both published and unpublished studies, eventually arguing “against some claims about the role of affect in moral judgments” (p. 518).

Still, there are further reasons to be wary of cherry picking and to appreciate the benefits of a more systematic approach to literature search and review. In particular, the crisis of findings’ reproducibility in psychology (Open Science Collaboration 2015; Pashler and Harris 2012) and other fields (Baker 2016) clearly highlights a number of relevant issues. For instance, several big-name findings and effects that have influenced philosophical discussions have recently failed to replicate, and it is important that replication studies are also considered. Notably, there has been a good deal of interest in philosophy in “stereotype threat”, even recently (e.g., Schouten 2015; McKinnon 2014). The original study, authored by Shih et al. (1999), found that Asian women performed worse on a math test when primed to think about their female identity, but better when they were primed to think about their Asian identity. Whilst widely disseminated in textbooks and papers, this finding has suffered from some failed attempts to replicate (e.g. Moon and Roeder 2014; Gibson et al. 2014). Consider, also, research on the “unconscious thought hypothesis”, which is often discussed in the philosophical literature (e.g., Frankish 2010; Levy 2014). Interestingly, findings suggesting the value of unconscious decision-making happen to have a record of failed replications (e.g. Calvillo and Penaloza 2009; Huizenga et al. 2012). Systematic methodologies offer important tools to portray a more accurate and balanced picture of science by encouraging the inclusion and discussion of replications as well.

Hopefully, combinations of narrative and systematic approaches will soon become more popular also within naturalistic philosophy. Many questions that have attracted the attention of philosophers, such as whether psychopathy increases propensity to engage in immoral behavior compared to subjects without it, or whether intelligence increases the likelihood of achieving good life outcomes in healthy subjects, can be tackled using the framework of systematic reviews. Exploring whether conclusions typically reached via narrative literature reviews would stand in light of the application of systematic tools seems an important task for naturalistic philosophers, and one in line with the mission of critically appraising scientific projects.

### The feasibility of systematic reviews

Having provided further support to the claim that naturalistic philosophers would greatly benefit from the application of systematic reviews, this section comments further on the feasibility of systematic approaches. Specifically, one might argue that formats such as PICO are too narrow for the purposes of naturalistic philosophers. But there is no compelling reason to consider systematic methodologies to be unfeasible.

Philosophers should not necessarily apply the PICO model previously described. The PICO model could be reasonably applied in those cases in which philosophers refer to experimental methods that use comparisons and focus on specific outcomes, but it fits less nicely qualitative research, where the latter might still be of interest to some empirically minded philosophers of mind who are looking for evidence coming from interviews to inform their analyses. For such cases, less strict formats of systematic reviews, such as those discussed in the previous section, seem to be preferable. Further, the very fact that systematic methodologies for search and review have already been introduced in some areas of the humanities and social sciences seems to speak in favor of the feasibility of the proposal made in this paper (Strech et al. 2008).

In fact, there are several possible applications of systematic methodologies that would depart from the PICO model. One natural application of systematic reviews is to explore the definitions and operationalizations of various concepts in relevant literatures. For instance, consider Machery’s (2009) challenge to the view that the concept of “concept” has been used in the same sense in philosophy and in cognitive science. This is one instance in which definitions and operationalisations of the concept could be fruitfully explored in a systematic way. But there are certainly many other possible applications. For instance, consider research on confabulatory phenomena, which were originally discussed in the context of patients with Korsakoff syndrome with severe amnesia. When asked what they did on a particular day, they would report as memories events that either did not happen or had happened much earlier in the patient’s life (e.g., Berlyne 1972). Some philosophers have recently criticized definitions of confabulation currently available in the scientific literature (e.g., Hirstein 2005; Robins 2016), but attempts to improve or reform the definitions used in a particular scientific literature seem to assume an accurate characterization of the ways in which the relevant phenomenon has been defined in the literature. As it turns out, the latter task is descriptive and well served by using systematic methodologies. Notably, systematic reviews of definitions seem to be one particular application of systematic reviews that might be of great interest and benefit research even beyond naturalistic philosophy.

Whilst systematic methodologies can indeed be successfully applied by philosophers, this is not to deny that some changes might be required in order to adapt the systematic approach to the field of philosophy, which is also interested in subject areas that are characterized by less rigid terminology than that used, for instance, in biomedicine (making comprehensive searching more challenging). More precisely, traditional systematic reviews prefer databases that include a wide range of publications of clinical trials, such as MEDLINE and EMBASE, and usually deal with issues (such as specific diseases and interventions) and study designs (such as randomized controlled trials) that correspond well to the controlled vocabulary of such databases. Research of relevance to naturalistic philosophers is often indexed in databases other than MEDLINE and EMBASE, and because of the heterogeneity of the search terms that are relevant for naturalistic philosophy and are used by different databases, search algorithms for systematic reviews by naturalistic philosophers have to be adapted to the databases’ vocabulary to enhance the sensitivity and specificity of literature searches.

Still, whilst defining the best search strategies and most suitable databases are important practical issues, these certainly should not and do not only concern philosophers. In several disciplines there are ongoing discussions about the most suitable databases for systematic reviews (Bramer et al. 2013, 2016; Gehanno et al. 2013; Martin-Martin et al. 2017; Vassar et al. 2017). Most likely, the ideal combination of databases that naturalistic philosophers should use will depend on the specific question they address. For instance, empirical papers published in philosophy journals and articles published in social sciences journals might not be displayed in databases such as *PubMed* and *Scopus*.

There are other important questions that naturalistic philosophers will need to address. For instance, how will a systematic review include or exclude studies based on quality? Would it be based on sample size or on *p* values? This is an important and burning question, for instance, in contemporary debates in psychology, which stress the need to improve statistical practices. Clearly, there can be disagreements on the choices made by authors with regard to these issues, but by making criteria explicit authors make themselves more easily accountable for their choices and favor methodological transparency and awareness of the importance of assessing the quality of the studies rather than accepting empirical conclusions at face value. The upshot of this section is that there are not obvious reasons why naturalistic philosophers should not adopt systematic approaches to literature search and review.

### Objection

Yet, a possible objection here is that naturalistic philosophers should let scientists review empirical literature systematically, and simply rely on the results of their systematic analyses. There are some problems with this claim, though. First, this rejoinder would still acknowledge that philosophers should pay close attention to results obtained via systematic methods, and just rejects the claim that philosophers should actively deploy such methods. In other words, philosophers should still appreciate that systematic reviews would constitute a privileged source of evidence to use in their philosophical work. Second, it also seems that by engaging directly with systematic methodologies, philosophers would disengage in part from the agenda of particular sciences, and would be able to contribute to redirect it towards topics that are especially important to philosophers. More precisely, there are plenty of topics that are of great interest to philosophers and that could be target of systematic methodologies. Consider a naturalistic philosopher interested in exploring whether conscious decision-making leads to better outcomes than non-conscious one: being able to properly apply systematic tools would greatly help her in her philosophical work. Instead, by relying passively on the syntheses provided by scientists, philosophers would risk failing to adequately answer questions that have been traditionally central in their disciplines. Further, experimental philosophers have also produced empirical work themselves, and they should be able to review their findings systematically, instead of expecting non-philosophers to accomplish the task for them.

### Practical recommendations

The case for systematic methodologies made in this paper has several noteworthy and direct implications for research, publishing and teaching practices in the philosophical community. It is helpful to widely underscore the importance of using objective and transparent procedures during search and review to authors, readers, and other stakeholders in the philosophical community. In particular, journal editors should consider updating their journals’ instructions for authors, which are the main way of communication between researchers, publishers and journal editors and serve as a readily available tool for reaching potential authors. Clearly written instructions may provide assistance throughout the whole process of manuscript preparation and it is a journal’s obligation to update instructions and inform authors about editorial policies, manuscript preparation preferences and requirements of accompanying documents for each submission (Gasparyan et al. 2014; Horvat et al. 2016). Journals’ editors should consider revising such guidelines to both emphasize the importance of transparent reporting and refer to external guidance on the best practices to conduce literature reviews and report results, such as the PRISMA Statement (http://www.prisma-statement.org). For instance, interested authors can find there a template for flow diagrams of the literature searching and sifting process. These diagrams provide the readers with a thorough and rapid presentation of everything authors did and why they did.

Further, journal editors can also encourage authors to release their datasets as part of a more general commitment to openness and reproducibility of findings. For instance, the Open Science Framework (http://osf.io/) offers resources for research collaboration, including the storage of documents and data. Prospective authors of systematic reviews could also be encouraged to pre-register their systematic review, closely pre-specifying the review design and criteria. As previously discussed, in the same way as it occurs with any prior preparation of an empirical study, in which its development should be preceded by a clearly defined question, an appropriately formulated problem and some background that justifies it, before embarking on the difficult task of preparing and publishing a systematic review, it is recommended to define the problem to be addressed and the specific aims and strategies that will guide the process. Planned systematic reviews can also be pre-registered, and support for study pre-registration is increasing: websites such as the Open Science Framework (http://osf.io/) and AsPredicted (http://AsPredicted.org/) offer services to preregister various kinds of studies. For instance, registered reports have been adopted by over 70 journals, covering a wide range of life, social and physical sciences (http://cos.io/rr/#journals). Importantly, journals can do more than merely advise on best practices and refer to external sources. For instance, they can provide incentives to more objective and transparent practices by acknowledging objective and open practices with badges in publications. This has now become more common in fields such as psychology. In addition, journals in the field of philosophy could also consider introducing a new article type, i.e. systematic review.^8^

Finally, it is also advisable to introduce training in systematic methodologies in educational contexts, in an attempt to equip students with tools and guidelines to better navigate extensive and growing bodies of literature. Interestingly, there have been some recent discussions on whether curricula in philosophy should now be updated to include also training in statistics, given that a growing number of philosophers are now reading and assessing empirical material (Knobe 2016).^9^ It is also the case, however, that providing training on how to apply systematic reviews and discussing their virtues and limitations will also greatly improve philosophical education, resulting in philosophers being better positioned at using and discussing empirical literature.

## Conclusion

In summary, this essay has attempted to highlight and discuss some overlooked problems with the methodology of naturalistic philosophy and to point to solutions that might help overcome them. More precisely, it has firstly been stressed that naturalistic philosophers have not adequately reflected on the obvious and yet important fact that literature search and review are likely to be affected by widespread and systematic biases. This has been shown to be highly worrying, as naturalistic philosophers do not typically philosophize in a vacuum, and in fact seem to rely on literature search and review in a number of ways and for several purposes. The suggested solution to tackle these problems comes from scientific disciplines. Whilst naturalistic philosophers have recently started to look at methods and tools from the sciences to expand their methodological toolkit and offer philosophy better chances of accomplishing its goals, it turns out that the tradition of systematic reviews of literature from scientific disciplines has been unduly neglected. But systematic reviews are important tools that minimize bias and allow for reproducibility and transparency. The upshot of this investigation is that, if naturalistic philosophers wish to reduce bias in philosophy, as it is here assumed that they should, they should consider ways to supplement their traditional tools for literature search and review by including systematic reviews.

## Footnotes

1 I wish to thank an anonymous reviewer for bringing my attention to this important aspect.

2 I wish to thank an anonymous referee for this journal for helping me clarify these points.

3 In other areas of philosophy, one could argue that things stand differently. For instance, John Passmore writes that “philosophers often seem to feel that they have a positive duty to conceal the origins of their thought. Alone in this—or in company only with journalists—they write articles which are wholly devoid of footnotes, which make no reference whatever to the previous literature on the same subject (1965, p. 4). Ludwig Wittgenstein, for example, provided us with one example of such attitude. In the preface of his *Tractatus logico-philosophicus*, he wrote: “How far my efforts agree with those of other philosophers I will not decide. Indeed what I have here written makes no claim to novelty in points of detail; and therefore I give no sources, because it is indifferent to me whether what I have thought has already been thought before me by another” (1922, p. 27).

4 (http://wokinfo.com/essays/impact-factor/).

5 The importance of the topic of discrimination against authors has recently gained importance and attention in philosophy: http://www.diversityreadinglist.org.

6 Also, Mercier and Sperber (2011) argue that biases such as confirmation can be adaptive as truth might not be the relevant goal in many conversational contexts. Still, in case of scholarly research, relevant goals seem to be epistemic in nature.

7 Hence, objections to systematic reviews which critique their application of statistical methods for evidence aggregation will not be discussed here (e.g., Stegenga 2011), as the use of meta-analytic tools is not essential to the argument made here.

8 This might be of special interest to journals specialized in work at the interface of philosophy and science. Systematic reviews could also be included in new editorial projects. For instance, philosopher De Cruz has recently made a case for the importance of launching a journal in experimental philosophy: http://philosopherscocoon.typepad.com/blog/2016/04/journal-of-experimentalphilosophyexpressions-of-interest.html . A journal specialized in such area, alongside many others with interests in naturalistic philosophy, would greatly benefit from the introduction of a new article type.

9 Cf. http://dailynous.com/2016/05/16/formal-methods-training-for-philosophy-graduate-students-guest-post-by-joshua-knobe/.
